# Exploratory Analysis to Predict Optimal Tumor Burden for Starting Lenvatinib in Patients With Radioiodine-Refractory Differentiated Thyroid Cancer

**DOI:** 10.3389/fonc.2021.638123

**Published:** 2021-07-08

**Authors:** Chiaki Suzuki, Naomi Kiyota, Yoshinori Imamura, Hideaki Goto, Hirotaka Suto, Naoko Chayahara, Masanori Toyoda, Yasuhiro Ito, Akihiro Miya, Akira Miyauchi, Masanori Teshima, Naoki Otsuki, Ken-ichi Nibu, Hironobu Minami

**Affiliations:** ^1^ Department of Medical Oncology/Hematology, Kobe University Graduate School of Medicine, Kobe, Japan; ^2^ Department of Otolaryngology-Head and Neck Surgery, Graduate School of Medicine, Kyoto University, Kyoto, Japan; ^3^ Kobe University Hospital Cancer Center, Kobe, Japan; ^4^ Department of Surgery, Kuma Hospital, Kobe, Japan; ^5^ Department of Otolaryngology-Head and Neck Surgery, Kobe University Graduate School of Medicine, Kobe, Japan; ^6^ Kindai University Faculty of Medicine, Department of Otolaryngology, Osaka, Japan

**Keywords:** radioiodine-refractory differentiated thyroid cancer (RR-DTC), multi-target kinase inhibitors (mTKIs), lenvatinib, long-term responders, maximum shrinkage of tumor burden

## Abstract

**Background:**

We previously reported that a high tumor burden is a prognostic factor based on an analysis of 26 patients with radioactive iodine-refractory differentiated thyroid cancer (RR-DTC) who were treated with lenvatinib. However, the optimal tumor burden for starting lenvatinib still remains to be defined. The aim of this retrospective study was to further explore in the same patient cohort the optimal timing for the start of lenvatinib by focusing on the pre- and post-treatment tumor burden.

**Methods:**

The 26 patients were treated with lenvatinib from 2012 to 2017. We explored the optimal timing for the start of lenvatinib by comparing the characteristics of long-term responders who were defined as patients with progression-free survival ≥ 30 months and non-long-term responders.

**Results:**

Long-term responders had a smaller post-treatment tumor burden at maximum shrinkage than non-long-term responders. Further, post-treatment tumor burden had a strong linear correlation with baseline tumor burden. We created an estimation formula for baseline tumor burden related to prognosis, using these regression lines. Patients with a sum of diameters of target lesions < 60 mm or maximum tumor diameter < 34 mm at baseline were estimated to have significantly better survival outcomes.

**Conclusions:**

We found a strong linear correlation between pre- and post-treatment tumor burden. Our results suggested a cut-off value for baseline tumor burden for long-term prognosis among patients treated with lenvatinib.

## Introduction

The most common type of thyroid cancer is differentiated thyroid cancer (DTC). Traditional treatment for DTC besides surgery includes radioactive iodine (RAI) and thyroid-stimulating hormone (TSH) suppression therapy. Most patients with DTC have a good prognosis, and even patients with metastatic disease can be cured by RAI. However, a small number (5–10%) will develop advanced disease, which becomes refractory to further radioiodine (1). Prognosis of RAI-refractory differentiated thyroid cancer (RR-DTC) is poor: 10-year survival in patients with metastatic DTC is only 10%, versus 60% in those who retain RAI avidity (2). One treatment option for patients with RR-DTC is multi-target kinase inhibitors (mTKIs). In the DECISION trial for RR-DTC, sorafenib showed a significant improvement in progression-free survival (PFS) (3), while in the SELECT trial, a phase 3 study in patients with RR-DTC, lenvatinib significantly improved PFS in patients with RR-DTC (4). Nevertheless, these trials did not demonstrate a significant improvement in overall survival (OS). In addition, the considerable toxicities associated with these mTKIs are of concern for patients with RR-DTC (3, 4): although manageable with treatment interruption and dose reduction, these toxicities still affect the quality of life (QOL) of the patients. Indeed, lenvatinib was discontinued in 14.2% of the patients in the SELECT trial and sorafenib in 18.8% in the DECISION trial (3). For these reasons, the optimal time at which to initiate treatment with mTKIs in patients with RR-DTC remains controversial.

Schlumberger et al. recommended that the decision to initiate systemic treatment should be based on several parameters, including tumor burden, disease progression, symptoms, and a high risk of local complications (5). We previously analyzed the prognostic factors including tumor growth rate, tumor burden and tumor-related symptoms for RR-DTC patients treated with lenvatinib and found that high tumor burden and tumor-related symptoms at baseline were independent prognostic factors (6). To date, however, no established criteria for the initiation of mTKIs in patients with RR-DTC have been established.

Here, we further explored in the same patient cohort the optimal baseline tumor burden at which to initiate lenvatinib by focusing on the pre- and post-treatment tumor burden.

## Materials and Methods

### Patients

The patients included in this analysis are the same 26 patients that were reported in our previous report (6). All had RAI-refractory disease, as defined earlier (4, 7). This retrospective study was approved by the appropriate institutional review board of Kobe University (ethical approval code: 180176). Data cut-off date for analysis was 31 July 2020. Eligibility criteria and dose modifications of lenvatinib were described in our previous report (6).

We categorized patients into two groups: long-term responders and non-long term responders. Long-term responders were defined as patients with PFS ≥ 30 months. Patients were also categorized into two groups according to median depth of response (DpR). DpR was defined as the percentage of maximum tumor shrinkage compared to baseline in the sum of diameters of target lesions (SumTLs) according to RECIST version 1.1 criteria (8).

### Evaluation of Tumor Parameters

Patient treatment responses were evaluated according to RECIST version 1.1 using whole-body computed tomography. We used the following tumor parameters: DpR, SumTLs, maximum tumor diameter of target lesion (MaxTL), the depth of size of MaxTL (DpS-TL) and SumTLs (DpS-Sum), thyroglobulin doubling time (Tg-DT), tumor diameter doubling times (TDT), and tumor growth slope (TGS).

SumTLs and MaxTL were defined as the target lesions except for bone metastases, longest diameters for non-nodal lesions with a longer axis of ≥ 10 mm and nodes with a short axis of ≥ 15 mm as per RECIST 1.1. DpS-TL and DpS-Sum were defined as the maximum shrinkage values of MaxTL and SumTLs during the clinical course.

We calculated Tg-DT and TDT using The Doubling Time and Progression Calculator (http://www.kuma-h.or.jp/english/), based on a concept previously reported by Miyauchi et al. (9). TGS is a parameter of tumor growth rate between two time points, and hence higher TGS means a higher tumor growth rate. TGS was calculated using a formula we described previously (6).

### Statistical Analyses

PFS was calculated as time from the date of diagnosis to documented disease progression, or death from any cause. OS was calculated as time from the date of diagnosis until death from any cause. Patients were censored at the time they were last known to be alive. PFS and OS were evaluated using the Kaplan–Meier method and differences between covariates were evaluated using the log-rank test. Differences in characteristics between outcomes in PFS and response were analyzed for significance using the chi-squared test for categorical values, t-tests and the Mann-Whitney U test for continuous variables. Receiver operating characteristics (ROC) curve was generated and the area under the curve [and its 95% Confidence Interval (CI)] was constructed to determine cut-off values of DpS-TL and DpS-Sum that yielded joint maximum sensitivity and specificity. Univariate linear regression was used to assess the association between the pre- and post-treatment tumor burden.

All data were analyzed with the JMP14 statistical software program (SAS Institute, Inc., Cary, NC, USA). Statistical significance was determined by a p-value below 0.05.

## Results

### Characteristics of the Study Population

Median follow-up period of the 26 patients was 36.8 (range, 1.3 to 84.8) months. Baseline characteristics of all patients are shown in **Table 1**. Eleven patients (42.3%) were identified as long-term responders. Long-term responders were less likely to have tumor-related symptoms (27.3% *vs*. 46.7%, p<0.01) than non-long-term responders. Other patient characteristics, including age, gender and comorbidities, did not differ significantly between the two groups.

**Table 1:** Patient demographics (N=26) .

Characteristic	Median or Number of patients (range or %)		
	Long-term responders (N=11)	Non-long-term responders (N=15)	p-value
**Age (years)**	65 (30- 80)	63 (39- 83)	0.67
**Gender**			
**Male**	3 (27.3%)	5 (33.3%)	0.74
**Female**	8 (72.7%)	10 (66.7%)	
**ECOG performance status**			
**0-1**	10 (90.9%)	13 (86.7%)	0.74
**2-3**	1 (9.1%)	2 (13.3%)	
**Tumor-related symptoms**			
**Yes**	3 (27.3%)	7 (46.7%)	<0.01
**No**	8 (72.7%)	8 (53.3%)	
**Histology**			
**Papillary carcinoma**	8 (72.7%)	11 (73.3%)	0.97
**Follicular carcinoma**	2 (18.2%)	3 (20.0%)	
**Poorly differentiated carcinoma**	1 (9.1%)	1 (6.7%)	
**Number of metastatic sites**			
**≤ 2**	10 (90.9%)	9 (60.0%)	0.08
**≥ 3**	1 (9.1%)	6 (40.0%)	
**Sites of metastasis**			
**Lung metastasis**	11 (100%)	15 (100%)	-
**Lymph nodes metastasis**	6 (54.5%)	12 (80.0%)	0.16
**Bone metastasis**	2 (18.2%)	7 (46.7%)	0.13
**Liver metastasis**	0 (0.0%)	2 (13.3%)	0.21
**Subsequent treatment**			
**Yes**	5 (45.5%)	4 (26.7%)	0.53
**No**	6 (54.5%)	11 (73.3%)	
**Sum of diameters of target lesions (mm)**	41.3 (26.0–137.3)	73.3 (26.4–229.2)	0.62
**Maximum tumor diameter (mm)**	22.9 (14.5–77.8)	37.1 (13.3–88.3)	0.14
**Thyroglobulin Doubling Time (year)**	0.62 (0.26–1.99)	0.60 (0.23–5.85)	0.80
**Tumor Doubling Time (year)**	0.66 (0.25–1.74)	0.41 (0.08–2.31)	0.60
**Pretreatment tumor growth slope**	4.1 (0.9–32.3)	4.9 (0.4–14.3)	4.9 (0.4–14.3)

### Efficacy

The median DpR was -41% for all patients receiving lenvatinib, and the median timing of first evaluation was 8 (interquartile range (IQR), 8–15) weeks. Median DpR in long-term responders and non-long-term responders was -50.8% (95% Confidence Interval (CI): -64.6– -37.1) *vs*. -33.9% (95% CI: -45.7– -22.1), respectively. Overall response rate was 76.9%. Median OS in long-term responders was not reached (95% CI: 80.3–NE) while that in non-long-term responders was 25.4 months (95% CI: 14.2–28.6).

### Relationship Between the Depth of Response (DpR), Survival and Baseline Tumor Burden

To examine the relationship between tumor response, survival and baseline tumor burden, we evaluated SumTLs, MaxTL and DpR. OS was longer in patients with DpR < -41% compared with > -41% (HR = 0.33 [95% CI: 0.10–0.95], p = 0.04) when patients were grouped using median DpR (-41%) as a cut-off. DpR showed a weak correlation with MaxTL (R-squared = 0.19, p = 0.03, **Supplementary Figure 1A**) and SumTLs at baseline (R-squared = 0.21, p = 0.02, **Supplementary Figure 1B**).

### Relationship Between Prognosis and Tumor Shrinkage

Spider plots of changes in MaxTL and SumTLs for all patients are shown in **Supplementary Figure 2**. From the spider plot analysis, baseline tumor burden appeared to be related with long-term tumor stabilization.

**Figure 1:**
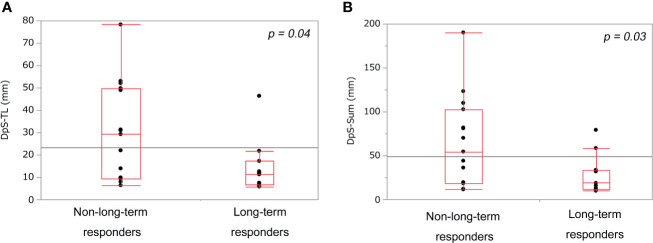
Comparison of post-treatment burden between non-long-term responders and long-term responders. DpS-TL **(A)** and DpS-Sum **(B)** among non-long-term responders and long-term responders. DpS-TL and DpS-Sum were significantly smaller in long-term responders than non-long-term responders. DpS-TL: the maximum shrinkage values of MaxTL (the maximum tumor diameter of target lesion) during the clinical course, DpS-Sum: the maximum shrinkage values of SumTLs (the sum of diameters of target lesions) during the clinical course.

DpS-TL and DpS-Sum during the clinical course were 13.3 mm (5.8–78.3) and 32.8 mm (9.9–190.1), respectively. Median DpS-TL and DpS-Sum in long-term responders and non-long-term responders was 14.0 mm and 27.4 mm vs. 30.1 mm and 64.9 mm, respectively. DpS-TL and DpS-Sum significantly differed between long-term responders and non-long-term responders (**Figures 1A, B**). The appropriate cut-off value of DpS-TL and DpS-Sum for long-term tumor response was 21.8 mm (area under the curve=0.76, sensitivity 90.9%, specificity 60.0%), and 33.7 mm (area under the curve=0.76, sensitivity 81.8%, specificity 66.7%), respectively, by ROC curve analysis (**Supplementary Figure 3**).

### Association of Survival Rate With DpS-TL and DpS-Sum

DpS-TL ≤ 21.8 mm was a significant positive prognostic factor for PFS (HR = 0.20 [0.07–0.57], p < 0.01) and OS (HR = 0.24 [0.08–0.66], p < 0.01) (**Supplementary Figures 4A, B**). DpS-Sum ≤ 33.7 mm was a significant positive prognostic factor for PFS (HR = 0.19 [0.06–0.54], p < 0.01) and OS (HR = 0.28 [0.10–0.77], p = 0.01). (**Supplementary Figures 4C, D**).

### Relationship Between the Pre-Treatment Tumor Burden and Maximum Shrinkage of Post-Treatment Tumor Burden

DpS-Sum strongly correlated with SumTLs (R-squared = 0.90) and DpS-TL correlated with MaxTL (R-squared = 0.84). Further, the relationship between these baseline and post-treatment parameters of tumor burden was linear:

SumTLs=24.3+1.04×DpS−Sum

MaxTL=11.2+1.02×DpS−TL

**Figure 2:**
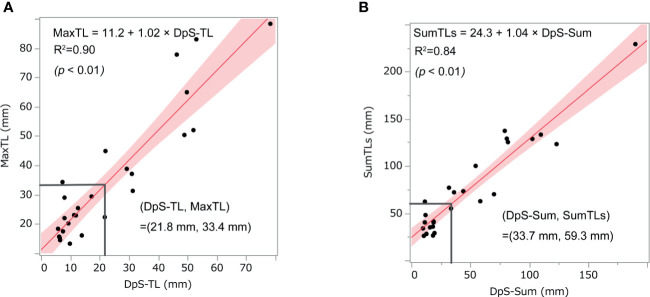
Relationship between post-treatment tumor burden and baseline tumor burden. The scatter diagrams show the relationship between **(A)** MaxTL and DpS-TL and **(B)** SumTLs and DpS-Sum. The straight line is the regression line and the shaded section shows the 95% confidence interval. MaxTL: the maximum tumor diameter of target lesion, DpS-TL: the maximum shrinkage values of MaxTL during the clinical course, SumTLs: the sum of diameters of target lesions, DpS-Sum: the maximum shrinkage values of SumTLs during the clinical course.

**Figure 3:**
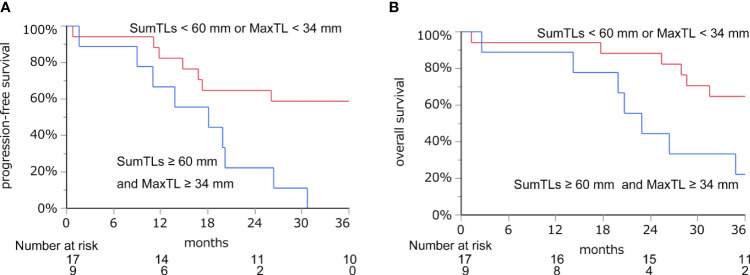
Progression-free survival **(A)** and overall survival **(B)** based on the cut-off value of SumTLs and MaxTL. Kaplan-Meier estimate of PFS and OS stratified by baseline tumor burden among patients treated with lenvatinib. MaxTL, the maximum tumor diameter of target lesion; SumTLs, the sum of diameters of target lesions.

With these regression lines, we estimated the optimal baseline tumor burden with which to start lenvatinib by assigning cut-off values of 21.8 mm for DpS-TL and 33.7 mm for DpS-Sum. As a result, SumTLs of 60 mm and MaxTL of 34 mm were estimated as optimal baseline tumor burden (**Figure 2**). SumTLs at baseline < 60 mm or MaxTL at baseline < 34 mm was a significant positive prognostic factor for PFS (HR = 0.27 [95% CI: 0.09–0.73], p=0.01) and OS (HR=0.31 [0.11–0.85], p=0.02) (**Figure 3**).

## Discussion

This study explored the optimal tumor burden at which to start lenvatinib in the treatment of RR-DTC in the same cohort with our previous report (6). As a result of this exploratory analysis, we found that post-treatment tumor burden at maximum shrinkage was associated with long-term tumor response and OS. Moreover, post-treatment tumor burden at maximum shrinkage (DpS-TL and DpS-Sum) had a strong linear correlation with baseline tumor burden (Max-TL and Sum-TLs). Consequently, we were able to estimate the optimal tumor burden at which lenvatinib should be initiated to achieve long-term response by a prediction model using a linear regression formula. From the prediction model, patients with baseline SumTLs < 60 mm or MaxTL < 34 mm were estimated to have significantly better survival outcomes; and these baseline tumor burdens might represent an appropriate time to initiate lenvatinib with the aim of a long-term response and survival.

RR-DTC is likely to show slow tumor growth. Sabra et al. reported that 39% of patients had pre-treatment TDT > 2 years (10). Guidelines from the American Thyroid Association recommend that patients with RR-DTC that is asymptomatic, stable, or minimally progressive who are not likely to develop rapidly progressive, clinically significant complications do not have indications for mTKIs, and that mTKIs should be considered in RR-DTC with metastatic, rapidly progressive, symptomatic, and/or imminently threatening disease not otherwise amenable to local control using other approaches (11). Moreover, from the NCCN Guidelines for Thyroid Carcinoma, mTKIs may not be appropriate for the patients with indolent disease or the asymptomatic patients since mTKIs will adversely affect the patient’s QOL (12). Indeed, in this present analysis, long-term responders were less likely to have tumor-related symptoms at baseline than non-long-term responders. Therefore, the initiation of mTKIs should be tailored to each patient to achieve stable disease, minimize progression and aid with symptom management.

Accordingly, indications for mTKIs should be considered with regard to information on tumor burden, tumor progression and threat to vital structures. Regarding the speed of tumor progression, from the inclusion criteria of the SELECT trial, disease progression within 12 months should be considered when starting mTKIs (4). Moreover, TDT was reported to have a potential role in the decision to start mTKIs (10, 13). In contrast, optimal baseline tumor burden in the start of mTKIs has not been investigated well.

With regard to the association between pre-treatment tumor burden and treatment outcomes with lenvatinib, some studies have suggested that tumor burden affects prognosis (6, 14, 15). We previously reported that a high tumor burden (SumTLs > 70mm) at baseline was a poor prognostic factor for PFS and OS in RR-DTC patients treated with lenvatinib (6). In a subgroup analysis of the SELECT trial, smaller baseline tumor (less than median baseline tumor size of 59.1 mm) was a favorable prognostic factor for PFS (HR = 0.61 [95% CI: 0.40–0.94], p=0.03) (14). In addition, patients with greater tumor size reduction during the first 8 weeks had significantly prolonged PFS, and the percentage tumor size reduction was associated with baseline tumor size (14). Our present exploratory analysis showed that post-treatment tumor burden has a strong linear correlation with baseline tumor burden. As a consequence, we were able to estimate the optimal tumor burden at which to start lenvatinib from the post-treatment burden using the suggested regression lines. From the regression lines, patients with baseline SumTLs < 60 mm or MaxTL < 34 mm were estimated to have significantly better survival outcomes.

Our study has several limitations. First, our formula was based on the assumption that almost all patients have uniform treatment effects. This assumption was itself based on the condition that we could expect a high response rate and good tumor reduction with lenvatinib. In fact, Robinson et al. reported a median maximum percentage change in tumor size of −42.9% among all patients and -51.9% among responders to lenvatinib in the SELECT trial (14), which were similar to our findings. Second, we could not evaluate QOL during the treatment and cost effectiveness. QOL and cost effectiveness are necessary for patients to live well during the treatment. It was reported that treatment with lenvatinib comparing with placebo was estimated an incremental cost-effectiveness ratio (ICER) of £65,872 (> £50,000) per quality-adjusted life-year gained (16). Although our optimal timing of the start of lenvatinib may achieve good prognosis, it may also increase ICER. Therefore, the assessment of QOL and cost effectiveness of mTKIs or novel molecular targeted agents for the patients with RR-DTC will be essential in the future prospective studies. Third, the study was conducted under a retrospective design in the same small patient cohort with our previous report (6). Although the selection process for eligible patients aimed to minimize selection bias, a degree of residual selection bias was inevitable. The retrospective, single-arm design restricted the results to candidate predictive factors only, and any decision on when to start lenvatinib should also consider such variables as tumor growth speed, imminent threat to vital structures and symptom deterioration, in addition to baseline tumor burden. Problems with the data arising from the small sample size may have caused greater bias in the estimated values than the model assumptions. Accordingly, our findings require validation in a prospective randomized trial with a larger number of patients.

We found a strong linear relationship between the pre- and post-treatment tumor burden in patients with RR-DTC. From the prediction model, patients with baseline SumTLs < 60 mm or MaxTL < 34 mm were estimated to have significantly better survival outcomes. These baseline tumor burdens might aid physicians and patients in clinically deciding an appropriate time for the initiation of lenvatinib, with the aim of long-term response and survival.

## Data Availability Statement

The raw data supporting the conclusions of this article will be made available by the authors, without undue reservation.

## Ethics Statement

The studies involving human participants were reviewed and approved by Kobe University, ethical approval code: 180176. The patients/participants provided their written informed consent to participate in this study. Written informed consent was obtained from the individual(s) for the publication of any potentially identifiable images or data included in this article.

## Author Contributions

Conceptualization, CS and KN. Methodology, CS and HG. Formal analysis, CS. Investigation, HS, NC, MTo, MTe, and NO. Data curation, CS, YIm, and HG. Writing—original draft preparation, CS. Writing—review and editing, NK, HG, YIt, and AMiya. Supervision, K-iN, AMiyauchi, and HM. Project administration, CS and KN. All authors contributed to the article and approved the submitted version.

## Conflict of Interest

NK has received grant support from Eisai, Inc.; research funding from Ono Pharmaceutical and Pfizer; and honoraria from Ono Pharmaceutical, Bristol-Meyers Squibb, Astra Zeneca, Eisai, Bayer and Merck Serono. K-iN has received honoraria from Bristol-Myers Squibb, Ono Pharmaceutical, Sanofi, Taiho, Nippon Shinyaku, MSD, Sanofi, Kyorin Tanabe-Mitsubishi, Rakuten Medical, Medtronic, Eisai, Astra Zeneca. HM has received research funding from Astellas, Bristol-Myers Squibb, Chugai, Dainippon Sumitomo Pharma, Eisai, Kyowa Hakko Kirin, Lilly Japan, Novartis, Ono Pharmaceutical, Sanofi, Taiho, Nippon Shinyaku, MSD, Boehringer Ingelheim, Daiichi Sankyo, Merck Serono, Takeda, Teijin Pharma, Yakult, Asahi Kasei and CSL Behring; and honoraria from Bayer, Bristol-Myers Squibb, Celgene, Chugai, Daiichi-Sankyo, Dainippon Sumitomo, Eisai, Janssen, Kowa, Kyowa Hakko Kirin, Lilly Japan, Merck Serono, Novartis, Ono Pharmaceutical, Otsuka, Pfizer, Sanofi, Shire, Taiho, Takeda and MSD; and filled a consulting/advisory role with Ono Pharmaceutical and Merck Serono.

The remaining authors declare that the research was conducted in the absence of any commercial or financial relationships that could be construed as a potential conflict of interest.

## Supplementary Material

Supplementary Figure 1 Relationship between baseline tumor burden and DpR. The scatter diagrams show the relationship between MaxTL and DpR **(A)**, and between SumTLs and DpR **(B)**. The straight line is the regression line, and the shaded section shows the 95% confidence interval. Time-dependent change for patients with RR-DTC treated with lenvatinib value. * DpR: depth of response/the percentage of maximum tumor shrinkage compared to baseline in the sum of diameters of target lesions, MaxTL: the maximum tumor diameter of target lesion, SumTLs: the sum of diameters of target lesion.

Supplementary Figure 2 Time-dependent change in MaxTL **(A)** and SumTLs **(B)** among patients with RR-DTC treated with lenvatinib. * MaxTL: the maximum tumor diameter of target lesion, SumTLs: the sum of diameters of target lesions.

Supplementary Figure 3 ROC curve for the association between DpS-TL **(A)**, DpS-Sum **(B)** and long-term responders. * DpS-TL: the maximum shrinkage values of MaxTL (the maximum tumor diameter of target lesion) during the clinical course, DpS-Sum: the maximum shrinkage values of SumTLs (the sum of diameters of target lesions) during the clinical course.

Supplementary Figure 4 progression-free survival **(A, C)** and Overall survival **(B, D)** based on the cut-off value of DpS-TL and DpS-Sum. Kaplan-Meier estimate of PFS and OS stratified by post-treatment tumor burden among patients treated with lenvatinib. * DpS-TL: the maximum shrinkage values of MaxTL (the maximum tumor diameter of target lesion) during the clinical course, DpS-Sum: the maximum shrinkage values of SumTLs (the sum of diameters of target lesions) during the clinical course.
