# Supplementary figures and images for: Exploratory Analysis to Predict Optimal Tumor Burden for Starting Lenvatinib in Patients With Radioiodine-Refractory Differentiated Thyroid Cancer
Source: Front Oncol. 2021 Jul 8;11:638123. doi: 10.3389/fonc.2021.638123 (PMC8298753; doi:10.3389/fonc.2021.638123)

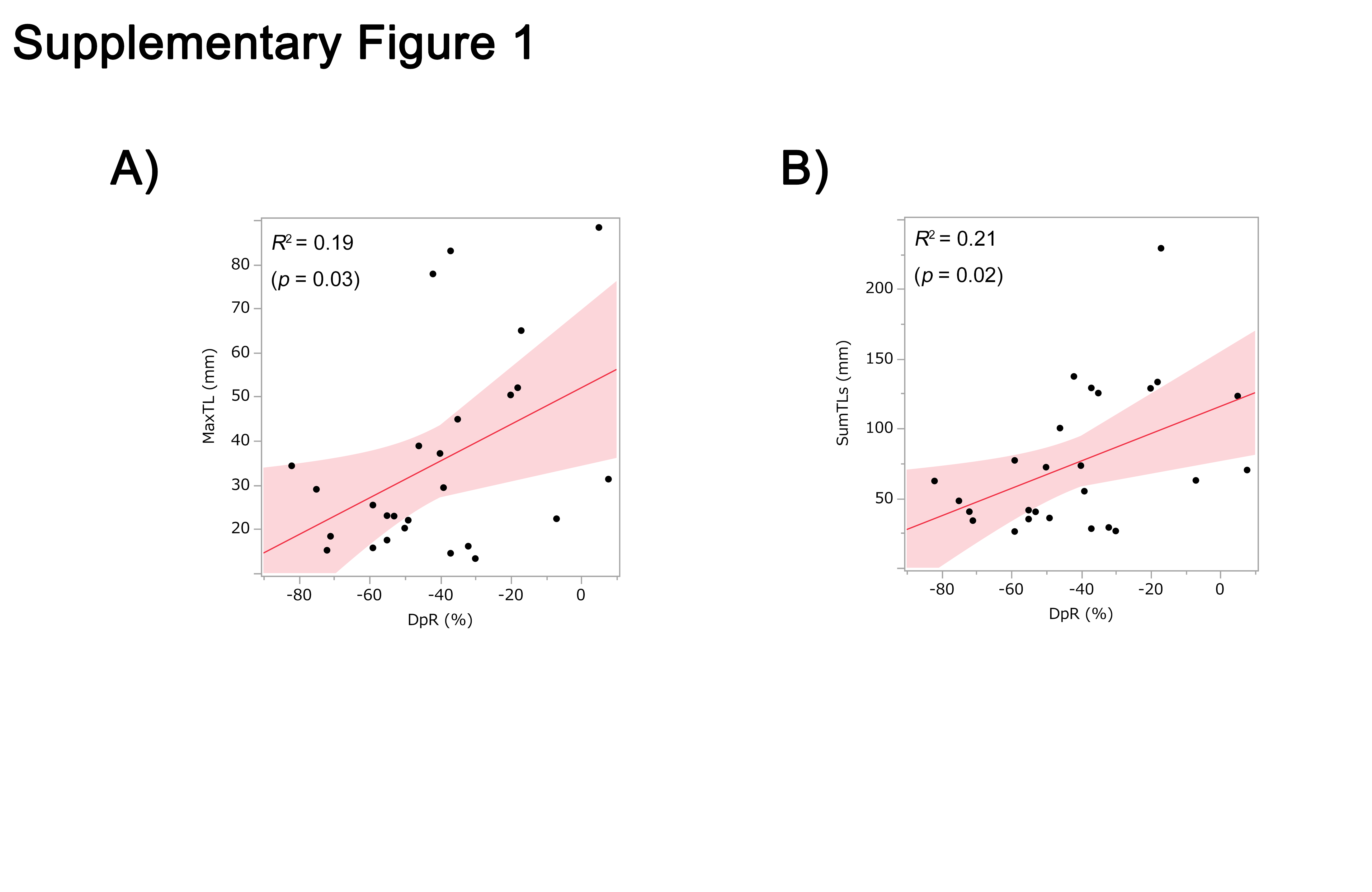

Supplement: Supplementary Figure 1 — Relationship between baseline tumor burden and DpR. The scatter diagrams show the relationship between MaxTL and DpR (A), and between SumTLs and DpR (B). The straight line is the regression line, and the shaded section shows the 95% confidence interval. Time-dependent change for patients with RR-DTC treated with lenvatinib value. * DpR: depth of response/the percentage of maximum tumor shrinkage compared to baseline in the sum of diameters of target lesions, MaxTL: the maximum tumor diameter of target lesion, SumTLs: the sum of diameters of target lesion. [file Image_1.tif]

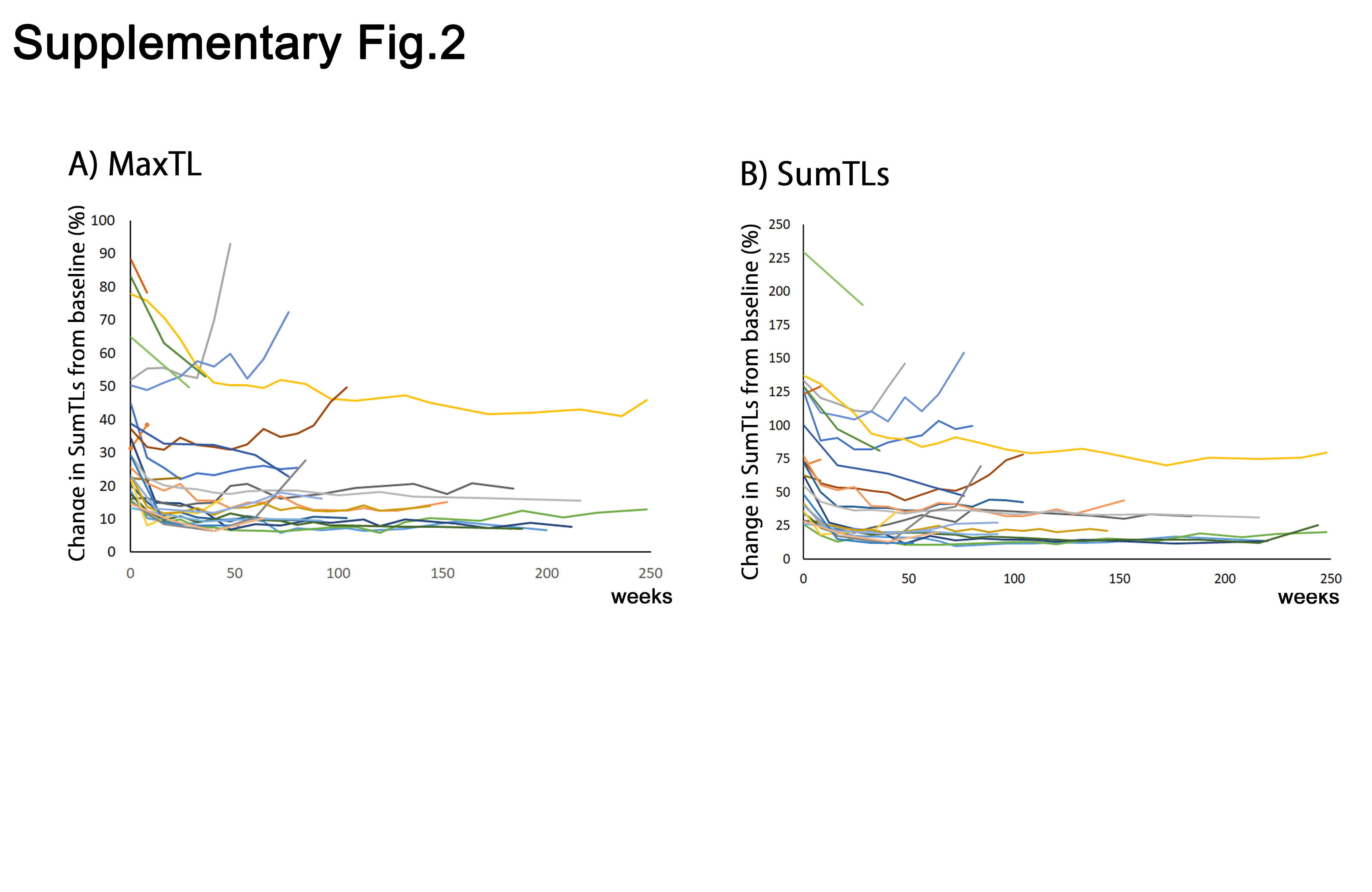

Supplement: Supplementary Figure 2 — Time-dependent change in MaxTL (A) and SumTLs (B) among patients with RR-DTC treated with lenvatinib. * MaxTL: the maximum tumor diameter of target lesion, SumTLs: the sum of diameters of target lesions. [file Image_2.tif]

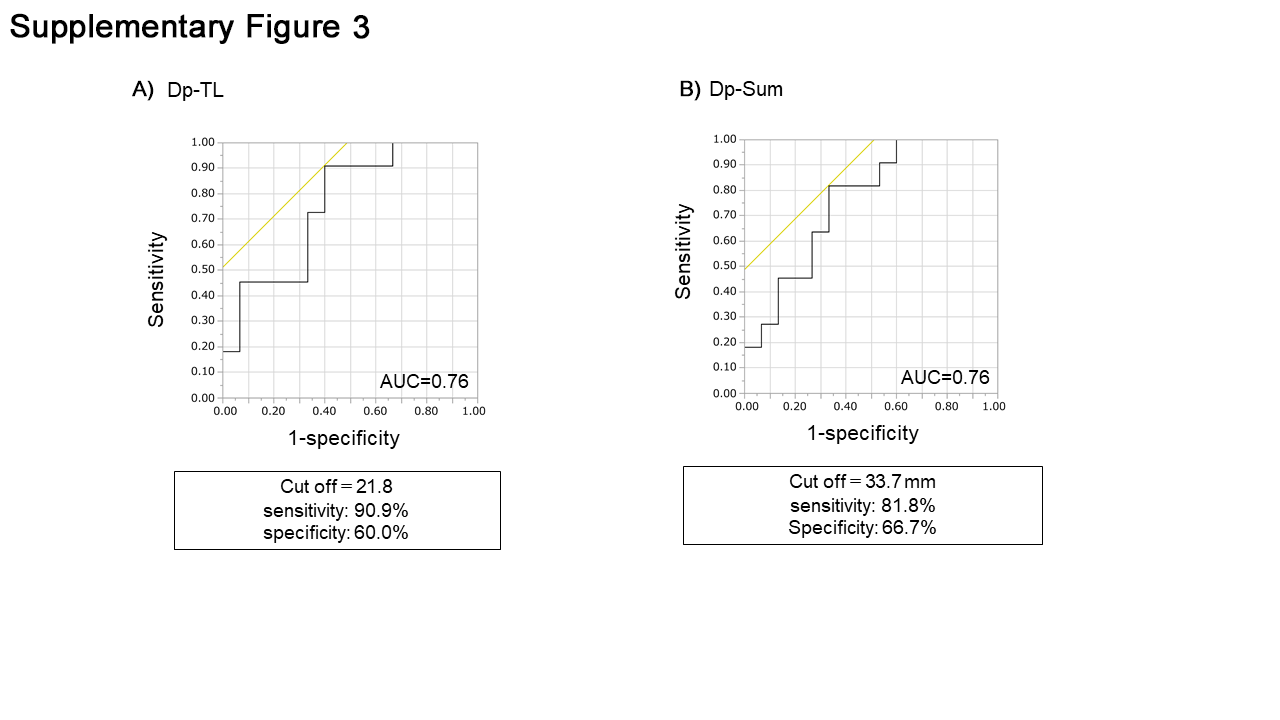

Supplement: Supplementary Figure 3 — ROC curve for the association between DpS-TL (A), DpS-Sum (B) and long-term responders. * DpS-TL: the maximum shrinkage values of MaxTL (the maximum tumor diameter of target lesion) during the clinical course, DpS-Sum: the maximum shrinkage values of SumTLs (the sum of diameters of target lesions) during the clinical course. [file Image_3.tif]

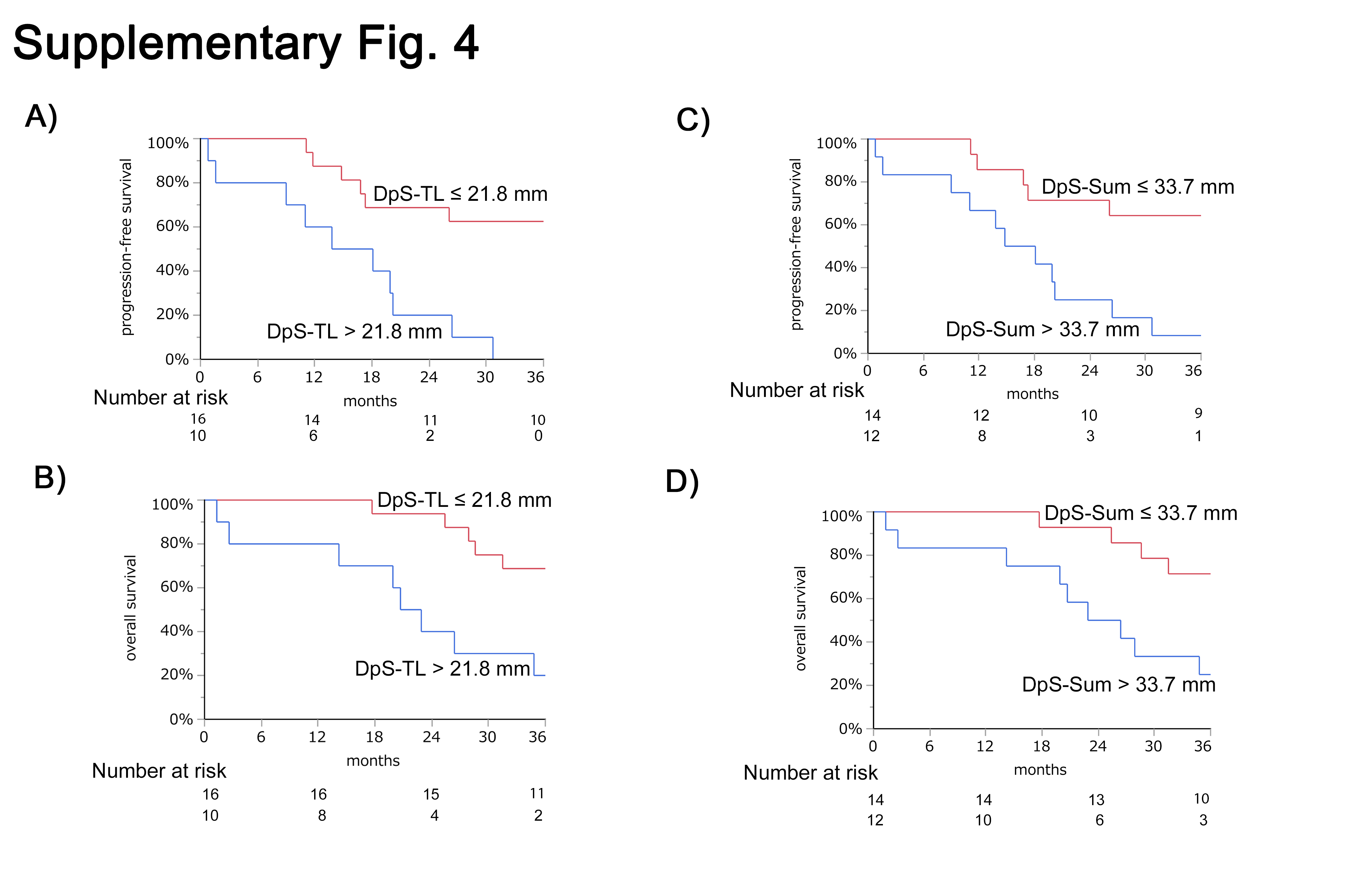

Supplement: Supplementary Figure 4 — progression-free survival (A, C) and Overall survival (B, D) based on the cut-off value of DpS-TL and DpS-Sum. Kaplan-Meier estimate of PFS and OS stratified by post-treatment tumor burden among patients treated with lenvatinib. * DpS-TL: the maximum shrinkage values of MaxTL (the maximum tumor diameter of target lesion) during the clinical course, DpS-Sum: the maximum shrinkage values of SumTLs (the sum of diameters of target lesions) during the clinical course. [file Image_4.tiff]
